# Proteome-wide comparison of tertiary protein structures reveals molecular mimicry in *Plasmodium*-human interactions

**DOI:** 10.3389/fpara.2023.1162697

**Published:** 2023-06-15

**Authors:** Viraj Muthye, James D. Wasmuth

**Affiliations:** ^1^ Faculty of Veterinary Medicine, University of Calgary, Calgary, AB, Canada; ^2^ Host-Parasite Interactions Research Training Network, University of Calgary, Calgary, AB, Canada

**Keywords:** molecular mimicry, malaria, plasmodium, AlphaFold, host-parasite interactions, structural comparisons

## Abstract

**Introduction:**

Molecular mimicry is a strategy used by parasites to evade the host’s immune system and facilitate transmission to a new host. To date, high-throughput examples of molecular mimicry have been limited to comparing protein sequences. However, recent advances in the prediction of tertiary structural models, led by Deepmind’s AlphaFold, enable the comparison of thousands of proteins from parasites and their hosts at the structural level, allowing for the identification of more mimics. Here, we present the first proteome-level search for tertiary structure similarity between proteins from *Plasmodium falciparum*, a malaria-causing parasite, and humans.

**Methods:**

We assembled a database of experimentally-characterized protein tertiary structures (from the Protein Data Bank) and AlphaFold-generated protein tertiary structures from *P. falciparum*, human, and 15 negative control species, *i.e*., species not infected by *P. falciparum*. We aligned human and control structures to the parasite structures using Foldseek.

**Results:**

We identified molecular mimicry in three proteins that have been previously proposed as mediators of *Plasmodium*-human interactions. By extending this approach to all *P. falciparum* proteins, we identified an additional 41 potential mimics that are supported by additional experimental data.

**Discussion:**

Our findings demonstrate a valuable application of AlphaFold-derived tertiary structural models, and we discuss key considerations for its effective use in other host-parasite systems.

## 1 Introduction

Parasites encounter host defenses at various points in their life cycle and employ a wide range of strategies for evading their host’s immune response and successfully transmitting to a new host (Chulanetra and Chaicumpa, 2021). These host-parasite interactions may be mediated by parasite-derived molecules—including proteins, lipids, sugars—that unexpectedly resemble host-derived molecules. This is termed ‘molecular mimicry’, which was originally defined as the sharing of antigens between parasite and host (Damian, 1964). One of the earliest reports of molecular mimicry came from the parasitic nematode *Ascaris lumbricoides*, which possesses A- and B-like blood group antigens in its polysaccharides (Oliver-González, 1944). The definition of molecular mimicry has adapted to keep up with molecular and genomic technologies and is now widely considered to similarity between proteins at the level of primary structure (amino acid sequence) and tertiary structure [summarized in (Tayal et al., 2022)]. An assumption is that molecular mimicry confers a fitness benefit to the pathogen. However, the term molecular mimicry is also used to explain the cross-reactivity between exogenous and self-peptides and is the theoretical framework for understanding autoimmunity (Getts et al., 2013). Related to both these definitions, molecular mimicry might also result in heterologous immunity, in which the infection from one parasite protects against infection by other parasites with similar antigenic molecules (Balbin et al., 2023).

Here, our focus is on molecular mimicry that confers a fitness advantage to the parasite, by either co-opting or disrupting the function of the mimicked host protein. Examples of molecular mimicry come from most branches of life. For instance, pathogenic bacterium *Escherichia coli* injects the TccP protein into host cells, which targets the polymerization of host actin. TccP contains multiple repeated motifs that mimic an internal regulatory element present in host N-WASP (neural Wiskott–Aldrich syndrome protein), which results in the activation of N-WASP (Sallee et al., 2008). This promotion of actin polymerization results in the creation of structures on epithelial cells that promote pathogen survival in the intestine. In another example, the myxoma virus decreases the number of activated macrophages by expressing its M128L protein on the host cell surface (Cameron et al., 2005). M128L shares significant sequence similarity with host CD47 and competes with it to bind with its receptor SIRPα. Within eukaryotic pathogens, the apicomplexan *Babesia microti* expresses the BmP53 protein which contains a domain that resembles thrombospondin (TSP1), a component of platelet cells (Mousa et al., 2017). The BmP53 TSP-1 is immunologically cross-reactive with human, and it is proposed that BmP3 helps cloak the extra-cellular stages from the immune system.

To the best of our knowledge, the first study to identify host-parasite molecular mimicry at a genome-scale across multiple species was by Ludin and colleagues (Ludin et al., 2011). They considered the protein sequences from eight species of eukaryotic parasites, the host (human), and seven non-pathogenic, eukaryotic, negative control species. Their approach identified multiple potential instances of mimicry in these parasites. For example, they detected a 14-amino acid motif in multiple PfEMP1 proteins in *Plasmodium falciparum* that was identical to the heparin-binding domain in human vitronectin, a protein with multiple roles in human including cell-adhesion. The approach was repeated to find ninety-four potential mimicry proteins in a tapeworm-fish system (Hebert et al., 2015). It was also adapted and expanded for use with 62 pathogenic bacteria and identified approximately 100 potential mimics (Doxey and McConkey, 2013). These approaches rely on two proteins sharing enough sequence similarity to be detected by the sequence alignment software, *e.g.*, BLAST (Altschul et al., 1990). However, these approaches depend on two proteins sharing a reasonably high level of local sequence similarity. For instance, several viruses express proteins with tertiary structure similarity, but undetectable sequence similarity, to human Bcl-2, and interfere with regulation of apoptosis (Kvansakul et al., 2007; Westphal et al., 2007). Similarly, in *Plasmodium falciparum*, a search of parasite proteins targeted to host extracellular vesicles revealed that at least eight shared unexpected and significant tertiary structure similarity with host proteins (Armijos-Jaramillo et al., 2021).

The opportunity to detect host-parasite mimicry at the level of tertiary structure has been limited by the number of available tertiary protein structures. Even for a parasite as important as *P. falciparum*, the protein databank (PDB) contains structures from less than 4% of the protein-coding genes in its genome (**Table 1**). We expect that most, if not all, other bacterial and eukaryotic pathogen species will have worse coverage. Proteome-wide searches for host-parasite molecular mimicry at the level of tertiary structure depended on *in silico* predictions that were of inconsistent quality (Armijos-Jaramillo et al., 2021). The prediction of tertiary protein structures from amino acid sequences has seen a much-publicized boon, in no small part to the development of AlphaFold (Ronneberger et al., 2021). In an early large-scale application, the AlphaFold Protein Structure Database (AFdb) provided tertiary structure predictions for 16 model organisms and 32 pathogen species of global health concern (https://alphafold.com). Complementing the release of AlphaFold was Foldseek, a novel approach to aligning tertiary protein structures (van Kempen et al., 2023). Comparisons showed that Foldseek was nearly 20,000 times faster than existing protein structure aligners while maintaining accuracy [but see (Holm, 2022)]. These two major advances in structural bioinformatics—AlphaFold and Foldseek—have empowered us to investigate the usefulness of using tertiary protein structures for identifying instances of host-parasite molecular mimicry.

**Table 1:** The number of tertiary protein structures for each species used in this study.

Category	Species	AlphaFold structures	PDB structures	Total number of tertiary structures
Control	*Arabidopsis thaliana*	20,460	4,560	25,020
Control	*Caenorhabditis elegans*	14,683	1,036	14,968
Control	*Candida albicans* SC5314/ATCC MYA-2876	4,777	285	5,813
Control	*Danio rerio*	19,541	1,026	20,567
Control	*Dictyostelium discoideum*	8,259	345	8,604
Control	*Drosophila melanogaster*	9,685	2,624	12,309
Control	*Escherichia coli strain* K12	4,220	11,242	15,462
Control	*Glycine max*	39,168	314	39,482
Control	*Methanocaldococcus jannaschii* strain DSM 2661	1,708	511	2,219
Control	*Mus musculus*	16,911	13,419	30,330
Control	*Oryza sativa subsp. japonica*	22,852	410	23,262
Control	*Rattus norvegicus*	16,555	7,512	24,067
Control	*Saccharomyces cerevisiae* strain ATCC 204508/S288c	4,745	15,228	19,973
Control	*Schizosaccharomyces pombe* strain 972/ATCC 24843	4,224	1,388	5,612
Control	*Zea mays*	24,721	456	25,177
Host	*Homo sapiens*	17,226	125,073	142,299
Parasite	*Plasmodium falciparum* isolate 3D7	2,909	1,417	4,326

Structures were downloaded from two sources - the AlphaFold Protein structure Database (AFdb) and the RCSB Protein Data Bank (PDB). These structures were filtered according to the steps outlined in Section 2.1.

Our parasitic species of study is *Plasmodium falciparum*. While our understanding of the mediators of host-parasite interactions is limited, as the leading cause of severe malaria in humans, *P. falciparum* might represent the current pinnacle of our knowledge. Furthermore, the protein tertiary structures are complemented with a broad range of curated -omics datasets available on PlasmoDB, which can help with candidate prioritization (Aurrecoechea et al., 2008; Amos et al., 2022). Proteins expressed by *P. falciparum* mediate interactions with its human host at multiple stages in its life cycle (Acharya et al., 2017). Molecular mimicry plays a role at both the liver and blood stages for immune evasion and cytoadherence. For instance, RIFIN, a prominent erythrocyte surface protein expressed by *P. falciparum*, binds to human LILRB1 which inhibits stimulation of the immune response. RIFIN does this by mimicking MHC Class I, the activating ligand of LILRBA (Harrison et al., 2020). Meanwhile, the circumsporozoite protein (CSP), which promotes invasion of human liver cells, has an 18 amino acid region that is similar to a cytoadhesive region in mammalian thrombospondin (Robson et al., 1988; Cerami et al., 1992).

In this study, our goal was to identify *P. falciparum* proteins which share tertiary structure similarity with human proteins but not detectable sequence similarity. First, we examined *P. falciparum* proteins which are known or have been implicated to directly interact with human biomolecules. We found new potential instances of molecular mimicry. Second, we extended our approach to consider all *P. falciparum* proteins and leveraged experimental datasets to filter the candidate mimics. Overall, our study highlights the advantages of using tertiary protein structures for identifying instances of molecular mimicry.

## 2 Materials and methods

### 2.1 Compiling the datasets of tertiary protein structures

We downloaded tertiary protein structures for *Plasmodium falciparum* 3D7 (parasite), human (host), and 15 negative control species, *i.e.*, species that are not infected by *P. falciparum* (**Tables 1**, **2**; **File S1**). Protein structures for these species were downloaded from two sources: 1) the RCSB Protein Data Bank (PDB) (experimentally determined protein structures, last accessed 06/16/2022), and 2) the AlphaFold Protein Structure Database (AFdb) (computationally predicted protein structures, https://alphafold.ebi.ac.uk/). The structures downloaded from both sources were processed before analysis (explained below).

**Table 2:** The number of tertiary protein structures from the AlphaFold Protein structure Database (AFdb) for each species used in this study.

Category	Species	AlphaFold structures (total)	AlphaFold structures (that passed filtering)	% AlphaFold Structures that pass the filtering
Control	*Arabidopsis thaliana*	27,434	20,460	74.58%
Control	*Caenorhabditis elegans*	19,694	14,683	74.56%
Control	*Candida albicans* SC5314/ATCC MYA-2876	5,974	4,777	79.96%
Control	*Danio rerio*	24,664	19,541	79.23%
Control	*Dictyostelium discoideum*	12,622	8,259	65.43%
Control	*Drosophila melanogaster*	13,458	9,685	71.96%
Control	*Escherichia coli strain* K12	4,363	4,220	96.72%
Control	*Glycine max*	55,799	39,168	70.19%
Host	*Homo sapiens*	23,391	17,226	73.64%
Control	*Methanocaldococcus jannaschii* strain DSM 2661	1,773	1,708	96.33%
Control	*Mus musculus*	21,615	16,911	78.24%
Control	*Oryza sativa subsp. japonica*	43,649	22,852	52.35%
Parasite	*Plasmodium falciparum* isolate 3D7	5,187	2,909	56.08%
Control	*Rattus norvegicus*	21,272	16,555	77.83%
Control	*Saccharomyces cerevisiae* strain ATCC 204508/S288c	6,040	4,745	78.56%
Control	*Schizosaccharomyces pombe* strain 972/ATCC 24843	5,128	4,224	82.37%
Control	*Zea mays*	39,299	24,721	62.90%

These structures were filtered according to the steps outlined in Section 2.1.

#### 2.1.1 Processing the PDB structures

**Figure 1:**
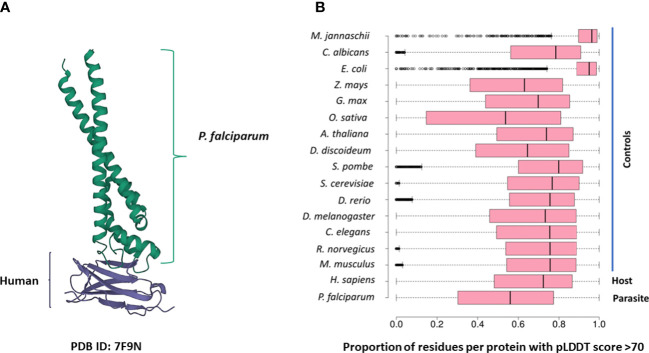
**(A)** The 7F9N protein tertiary structure from the RCSB Protein Data Bank. The chains from the *P. falciparum* RIFIN protein (RIF, PF3D7_1000500) are colored green and the chains from the human LAIR protein (LAIR1, Q6GTX8) are colored in purple. **(B)** Box-plot representing the overall prediction accuracy for each protein in the AFdb. Each point in this distribution represents the proportion of all residues with a pLDDT score above 70 for one AlphaFold structure. The median value is the parallel bar within the box. The limits of the box are the 25^th^ and 75^th^ percentiles, the whiskers extend 1.5 times the interquartile range, and the dots are the outliers.

Several PDB structures were composed of chains from multiple source organisms. We separated such structures into individual chains and extracted the appropriate chains corresponding to each species. For instance, the PDB structure 7F9N is composed of four chains (A to D), of which two chains (A and B) are from *P falciparum* Rifin (RIF, PF3D7_1000500) and two chains (C and D) are from the human leukocyte-associated immunoglobulin-like receptor 1 protein (LAIR1, Q6GTX8) (**Figure 1A**). We included only chains A and B for *P. falciparum*. Additionally, multiple structure chains were chimeric or ambiguous, *i.e.*, mapping to multiple source organisms. For instance, the PDB structure 4O2X has two chains (A and B) which map to both *P. falciparum* and *Escherichia coli* strain K12. Such chains were discarded to not confound downstream analysis.

#### 2.1.2 Processing the AlphaFold structures

AlphaFold assigns a score to each residue in the predicted structure called the ‘pLDDT’ score. This score is a measure of prediction confidence for that residue. The pLDDT scores range from 0 (low confidence of prediction) to 100 (high confidence of prediction). Regions with a pLDDT score above 90 are modelled with high accuracy, between 70-90 are modelled well, and 50-70 are modelled with low confidence. The AlphaFold database suggests that regions with pLDDT scores less than 50 should not be interpreted as this low score could be indicative of intrinsic protein disorder. For each AlphaFold structure, we calculated the proportion of the total residues with a pLDDT score of more than 70. We retained predicted structures with at least half the residues of the structure modelled with a pLDDT score above 70 (**Figure 1B**; **File S1**).

### 2.2 Identification of *Plasmodium falciparum* proteins known to interact with human molecules

We performed a literature survey to identify *P. falciparum* proteins that are known to interact with human molecules. We started with a review of *P. falciparum*-human protein interactions (Acharya et al., 2017). Then, we identified all abstracts on PubMed using the query [‘plasmodium falciparum’ AND ‘interact*’ AND ‘protein*’ AND ‘human*’] from 2017 to 2022. This resulted in 648 abstracts (as of 8/8/2022 1:36 PM). We read all 648 abstracts to identify the *P. falciparum* proteins of interest. The PlasmoDB ID for each protein was mapped to Uniprot IDs using PlasmoDB (Release 52, 30 August 2022). Three large gene families (PfEMP1, RIFIN, and STEVOR) play an important role in host-parasite interactions and pathogenesis in *P. falciparum.* Proteins belonging to these three families were downloaded from PlasmoDB.

### 2.3 Identification of sequence and structure similarity between *Plasmodium falciparum* and human proteins

#### 2.3.1 Analysis of protein sequence similarity

We analyzed sequence similarity between the proteins from *P. falciparum*, human, and 15 negative control species. We determined sequence similarity using three pairwise alignment search tools. SSEARCH36 implements the Smith-Waterman algorithm guaranteeing the optimal alignment. We used the following parameters for SSEARCH36 from Fasta36 ‘m 8 -s BL62 -f 12 -g 1’. BLASTP (Altschul et al., 1990) and DIAMOND (Buchfink et al., 2021) implement heuristic algorithms that are faster than SSEARCH36 but do not guarantee the optimal alignment. We used BLASTP with an e-value cut-off of 1e^-3^ and performed an ultra-sensitive DIAMOND BLASTP search with the same e-value cut-off. For both aligners, we searched using the BLOSUM45 and BLOSUM62 substitution matrices. All other parameters were left as default. Additionally, we used OrthoFinder version 2.5.4 to identify groups of orthologous proteins between all the 17 species used in this study, using DIAMOND as the aligner (Emms and Kelly, 2019). The results of this analysis were used to identify human proteins that had orthologs in only the other three vertebrates used in this study (mouse, rat, and zebrafish).

#### 2.3.2 Analysis of protein structure similarity

We aligned all *P. falciparum* structures to a database consisting of human structures and control structures. The structural aligner used was Foldseek v4 (easy-search -s 9.5 –max-seqs 1000). We used an e-value cut-off of 0.01. Foldseek was also used to visualize structural alignments using the option ‘format-mode 3’. Individual structures were visualized using EzMol (Reynolds et al., 2018). Additionally, we used Foldseek to calculate the TM-score of the aligned regions (–format-output ‘query, target, alntmscore’). While a structure might have more than half its residues with a pLDDT score of at least 70, it is possible that a region of poorly modelled residues in such structures could be aligned to another structure. Thus, we calculated the average pLDDT score of the aligned residues as well as the proportion of the aligned residues with pLDDT of at least 70. This was done to ensure that the regions of the predicted structures in the alignments were modelled well.

#### 2.3.3 Expression analysis

Expression analysis of *P. falciparum* proteins was carried out using the publicly available RNA-seq datasets available in PlasmoDB. We identified all *P. falciparum* proteins with expression in the 90^th^ percentile in at least one of the stages in the intra-erythrocytic life cycle (young ring 8 hpi, late ring/early trophozoite 16 hpi, mid trophozoite 24 hpi, late trophozoite 32 hpi, early schizont 40 hpi, schizont 44 hpi, late schizont 48 hpi, and purified merozoites 0 hpi) using data from (Wichers et al., 2019). We also identified all *P. falciparum* proteins with expression in the 90^th^ percentile in the ring and/or sporozoite stage using data from (Zanghì et al., 2018).

#### 2.3.4 Analysis of protein subcellular localization

To identify *P. falciparum* proteins carried in extracellular vesicles (EV), we applied an approach used in (Armijos-Jaramillo et al., 2021). In this study, we define a parasite EV protein as one identified in at least one of the three studies in the abovementioned study (Mantel et al., 2013; Abdi et al., 2017; Sampaio et al., 2018). We extracted information on subcellular localization of *P. falciparum* and human proteins from Uniprot and PlasmoDB.

## 3 Results

### 3.1 Assembling and filtering the datasets of crystallized and computationally predicted tertiary protein structures

We compiled tertiary protein structures from *Plasmodium falciparum* 3D7 (parasite), human (host), and 15 negative control species, those not infected by the parasite (**Tables 1**, **2**; **File S1**). These structures were downloaded from the RCSB Protein Data Bank (PDB) and the AlphaFold Protein Structure database (AFdb). All AlphaFold-generated structures were filtered using the pLDDT score, a per-residue metric of the confidence of prediction accuracy. Following the AlphaFold documentation, we considered structures to be high confidence if at least half their residues had a pLDDT score above 70. Through this filtering, we retained 56% of *P. falciparum* structures, 74% of human structures, and between 97% (*E. coli*) and 52% (*Oryza sativa*) for the control species (**Tables 1**, **2** and **Figure 1B**; **File S1**).

### 3.2 Investigating the effect of the source of tertiary structures on Foldseek alignments

We wanted to determine whether the source of the tertiary structure—crystallised (PDB) or computationally-predicted (AlphaFold)—affected the Foldseek search results. Following our filtering steps, 167 P*. falciparum* proteins were represented by structures from both PDB and AlphaFold and 159 aligned to at least one structure from the host and/or negative control species. For each of these 159 proteins, we compared the Foldseek results for their PDB and AlphaFold structures. For most of these proteins (107/159), the results for both PDB and AlphaFold queries agreed between 90 and 100%. For almost 10% of these proteins (14/159), the agreement between the results was less than 50% (**File S2**; **Figure S1**).

### 3.3 Structural analysis of parasite proteins experimentally known to interact with human proteins

We performed a literature review and identified 74 P*. falciparum* proteins that interact with human molecules at various stages in the parasite’s life cycle (**File S3**). We also included three large *P. falciparum* gene families which are thought to play a role in parasite virulence—PfEMP1 (61 proteins), RIFIN (158 proteins), and STEVOR (32 proteins) (**File S3**). Overall, 206 of these proteins were represented by at least one structure in our database of PDB and high-quality AlphaFold structures. To understand whether molecular mimicry plays a role in how these proteins interact with the host, we asked the question: do these 206 P*. falciparum* proteins share sequence and/or tertiary structural similarity with human proteins?

Of the 206 proteins, 171 did not have sequence or structure similarity to any human protein. We found that 31 of the remaining 35 P*. falciparum* proteins shared structural similarity to at least one human protein. Of these, three proteins aligned to human proteins which were restricted in vertebrates at the sequence-level (orthologs only in mouse, rat, and/or zebrafish). They were the parasite circumsporozoite protein (CSP, PF3D7_0304600) and two PfEMP1 proteins (PF3D7_0800100 and PF3D7_0617400). CSP was aligned to human thrombospondin (TSP1, P07996) (alignment TM-score greater than 0.6 for all alignments). Interestingly, as per previous sequence-based approaches, CSP mimics a cytoadhesive region in mammalian thrombospondin (Robson et al., 1988; Cerami et al., 1992).

**Figure 2:**
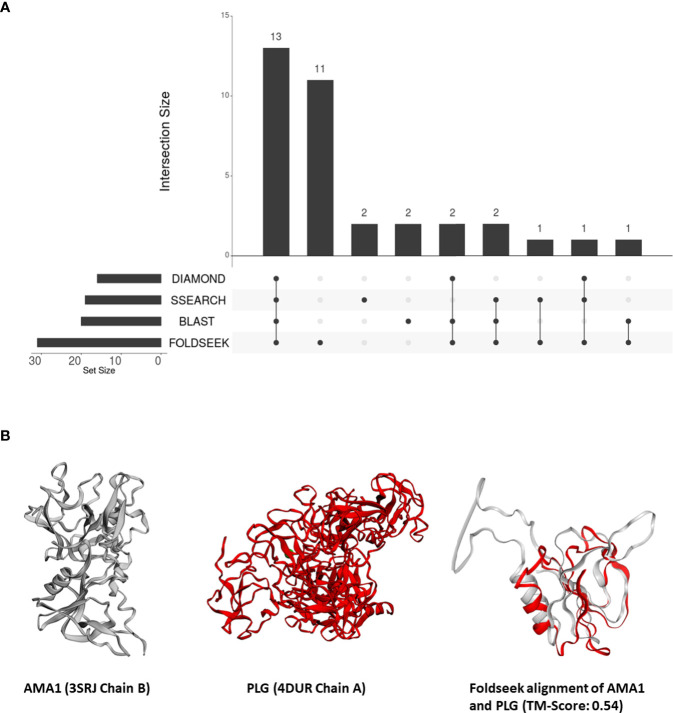
**(A)** An UpSet plot of the 206 parasite proteins that interact with human molecules and are represented by at least one structure in our database. The R package ‘UpSetR’ was used to generate the UpSet plot (Conway et al., 2017). This plot displays the number of these interacting parasite proteins aligned to human proteins by DIAMOND, BLAST, Foldseek, and/or SSEARCH36. **(B)** Tertiary structure alignments of the parasite AMA1 structure in grey (3SRJ chain B), the human PLG structure in red (4DUR chain A), and the region aligned by Foldseek.

From these 31 P*. falciparum* proteins which share structural similarity to at least one human protein, we removed all proteins which shared sequence similarity with human proteins – 18, 16, and 17 proteins that aligned to human proteins by BLASTP, DIAMOND, and SSEARCH36 respectively (**Figure 2A**). Interestingly, over one-third of these 31 P*. falciparum* proteins (11/31) did not have detectable sequence similarity to any human protein (**Figure 2A**). For these 11 proteins, we visually inspected their structural alignments with human proteins (**File S4**). The top human alignments for seven of these 11 proteins had a low TM-score (<0.2) and were not analysed further (**File S4**). Here, we present the biological relevance of their interactions for three of the remaining four proteins.

#### 3.3.1 P38

The 6-cysteine protein/merozoite surface protein P38 (P38, PF3D7_0508000) functions in red-blood cell invasion and binds to human glycophorin-A (GLPA, P02724) (Paul et al., 2017). P38 belongs to the 6-cysteine protein family and is expressed on the surface of the blood stages of the *P. falciparum* life cycle (Feller et al., 2013; Lyons et al., 2022). We found that P38 was structurally similar to a region containing an immunoglobulin domain in human titin (TTN, Q8WZ42). Specifically, the AlphaFold prediction for *P. falciparum* P38 was aligned to the human PDB structure 3LCY (‘Titin Ig tandem domains A164-A165’). The next match was the human receptor-type tyrosine-protein phosphatase F (PTPRF, P10586); P38 was structurally similar to a region containing two Ig-like domains. Ig-like domains are present in several adhesion proteins in human, where they play an important role in homophilic cell adhesion (Leshchyns’ka & Sytnyk, 2016). This suggests that P38, in addition to binding to GLPA, functions in adhesion owing to its similarity to the Ig-like domains.

#### 3.3.2 AMA1

The apical membrane antigen (AMA1, PF3D7_1133400) is a potential vaccine target (Remarque et al., 2008). Previous studies have suggested that the interaction between AMA1 and the *P. falciparum* rhoptry neck protein (RON) is essential for merozoite invasion (Srinivasan et al., 2011). AMA1 also interacts with the human erythrocyte membrane transport protein Kx protein (Kato et al., 2005). However, a later study demonstrated that AMA1 is involved in the attachment of the merozoite to the host cell, but is dispensable for cell invasion (Bargieri et al., 2013).

In our analysis, AMA1 was aligned to the PAN protein domain in human plasminogen (PLG, P00747) (top human Foldseek alignment based on bit-score) (**Figure 2B**). While this similarity could not be detected by sequence-similarity searches, domains I and II of *P. falciparum* AMA1 adopt a PAN domain fold (Bai et al., 2005). These PAN modules are involved in diverse protein-protein and protein-carbohydrate interactions (Tordai et al., 1999). Human PLG, possesses a signal peptide and, as per Uniprot, is secreted and localized to the cell surface. AMA1, on the other hand, is surface-exposed in sporozoites (Swearingen et al., 2016), and is also predicted to possess a signal peptide. Thus, we posit that merozoite attachment to human cells is mediated by its structural similarity to human plasminogen.

#### 3.3.3 CyRPA

The cysteine-rich protective antigen (CyRPA, PF3D7_0423800) interacts with two other parasite proteins (RH5 and Ripr) to form a complex on the surface of an invading merozoite (Ragotte et al., 2020). Understanding the similarity of CyRPA to human proteins is important because this complex is considered a potential vaccine target (Ragotte et al., 2020). CyRPA was represented by multiple structures from both the PDB and the AlphaFold Protein Structure Database and was similar to structures from several human proteins. The alignment with the best TM-score was to the region containing the EPTP protein domain (PF03736) in the human adhesion G-protein coupled receptor V1 protein (ADGRV1, Q8WXG9). This domain is likely a member of the 7-bladed beta-propeller fold (Scheel et al., 2002). The alignment with the second-best TM-score was to the human sialidase-1 protein. Both these results are interesting since CyRPA is known to form a 6-bladed beta-propeller fold that is similar to the sialidase fold, even though CyRPA has no sialidase activity (Favuzza et al., 2017).

In our earlier filtering step, we excluded AlphaFold structures if more than half of the predicted residues had low confidence (pLDDT<70). However, it is important to note that the retained structures may still contain regions with low confidence. The Foldseek algorithm considers the 3D neighbourhood of every residue, so we cannot mask out short regions of low confidence from the structural model directly. To address this, we checked the alignments of the four *Plasmodium* proteins described above and the aligned regions of three—AMA1, P38, and CyRPA—had a mean pLDDT greater than 70. Furthermore, the TM-scores for AMA1 and CyRPA was high (>0.5) and was above the noise cut-off for P38 (0.3) (**File S4**). Together this further supports the reliability of these alignments.

### 3.4 Large-scale analysis of the structural similarity between *Plasmodium falciparum* and human proteins

**Figure 3:**
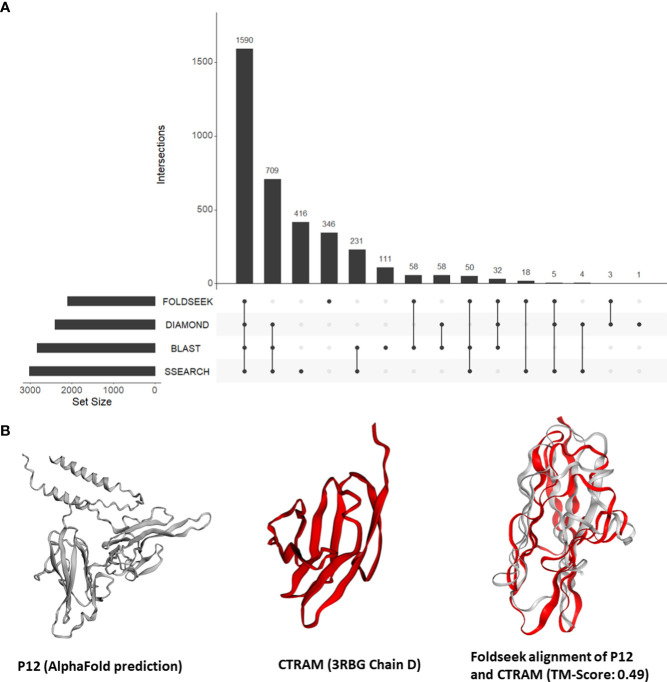
**(A)** An UpSet plot of all *Plasmodium falciparum* proteins which were aligned to human proteins by Foldseek, DIAMOND, BLASTP, and/or SSEARCH36. The R package ‘UpSetR’ was used to generate the UpSet plot (Conway et al., 2017). **(B)** Tertiary structure alignments of the parasite P12 structure in grey (AlphaFold prediction) and the human CTRAM structure in red (3RBG chain D), and the region aligned by Foldseek.

Our findings from the previous section demonstrated the effectiveness of our approach in identifying instances of unexpected structural similarities to human proteins that play a role in host-*Plasmodium* interactions. This motivated us to expand our approach and search all tertiary structures from *P. falciparum* against a database of 415,164 structures from human and 15 negative control species. Out of a total of 4,326 tertiary structures for *Plasmodium*, 3,649 (84%) aligned to a structure from either human or the negative control species. Out of the 3,350 (77%) could be aligned to a human structure, 59 structures aligned only to vertebrate proteins (human, mouse, rat, zebrafish) and 27 aligned to only mammalian proteins. Only eight structures aligned exclusively to a human structure. Nearly one-fifth of the *P. falciparum* proteins that shared structural similarity to human proteins (346 of 2,120) had no detectable sequence similarity to a human protein (**Figure 3A**; **File S5**). On average, 326 P*. falciparum* proteins had structural similarity but no sequence similarity to the control proteins.

To prioritise the proteins, we categorized them with available annotations from PlasmoDB and Uniprot. In category 1, we analysed the predicted function of the aligned human protein, where the top scoring alignment was with a human protein with a GO term of interest: ‘immune system process’, ‘cell adhesion’, ‘cytoskeleton’, and/or ‘signalling’. In category 2, we examined the likely export of the *P. falciparum* protein from the cell, where the *P. falciparum* protein was predicted to contain a signal peptide. Finally, category 3 was gene expression, where we selected *P. falciparum* proteins whose genes were expressed in the 90^th^ percentile in at least one of the human life cycle stages of the parasite—sporozoite and ring—or one of the intraerythrocytic stages (Zanghì et al., 2018; Wichers et al., 2019).

A total of 145 P*. falciparum* proteins could be placed in at least one of the categories, with 85 proteins in category 1, 41 in category 2, 76 in category 3, 31 in two categories, and 13 proteins in all three categories (**Figure S2**). The 44 P*. falciparum* proteins present in more than category represent instances of mimicry (**File S4**). Here, we present the biological relevance of a subset of these proteins.

First, we focused on the 13 P*. falciparum* proteins placed in all three categories (**File S4**). These 13 proteins include three known mediators of human-*Plasmodium* interactions mentioned in section 3.3 (CyRPA, P38, and AMA1). In addition, the *P. falciparum* 6-cysteine protein P12/merozoite surface protein P12 (P12, PF3D7_0612700) was structurally similar to the human cytotoxic and regulatory T-cell molecule (CRTAM, O95727) (alignment TM-score = 0.49) (**Figure 3B**). Interestingly, while the precise function of P12 is not known (Lyons et al., 2022), 6-cysteine proteins are one of the most abundant surface antigens in the *P. falciparum* have been attributed to virulence in related parasites (Wasmuth et al., 2012). P12 is localized on the surface of schizonts and merozoites. CRTAM is a host cell membrane protein and is involved in the development of cytotoxic CD4 and CD8 cells (Takeuchi et al., 2016). The expression of CRTAM increases during *P. falciparum* infection (Dooley et al., 2022). Furthermore, from StringDB, we found that eight of the 10 interacting partners of human CRTAM were mapped to ‘immune system process’ (GO:0002376) (**Figure S3**). Therefore, we posit that P12 mimics CRTAM to modulate the host immune system during infection.

We then expanded our focus to *P. falciparum* proteins found in two categories. The *P. falciparum* protein sporozoite surface protein 3 (SSP3, PF3D7_0812300) was aligned to human EGF-containing fibulin-like extracellular matrix protein 2 (EFEMP2, O95967) (alignment TM-score=0.58), with both proteins predicted to possess a signal peptide. EFEMP2 has been proposed to bind heparan sulfate (Djokic et al., 2013). This is interesting because multiple *P. falciparum* proteins, like the circumsporozoite protein (CS) (Frevert et al., 1993) and thrombospondin-related adhesive protein (TRAP) (Robson et al., 1995), are known to bind heparan sulfate. Our results suggest that SSP3 functions in targeting the sporozoites to the liver by mimicking the heparin-binding activity of EFEMP2.

Another *P. falciparum* protein was found to mimic a heparin-binding human protein. The uncharacterized *P. falciparum* protein (PF3D7_1118900), which possesses a signal peptide, was aligned to the region containing the MAM protein domain (PF00629) in human nephronectin (NPNT, Q6UXI9) (alignment TM-score=0.48). NPNT is a basement membrane protein which binds to heparin and basement membrane heparan sulfate proteoglycans through its MAM protein domain (Sato et al., 2013). Thus, our approach found a potential function for the uncharacterized *P. falciparum* protein. We propose that the uncharacterized *P. falciparum* protein mimics either the heparin-binding or the chondroitin sulfate-binding property of NPNT.

The *P. falciparum* glutaminyl-peptide cyclotransferase (PF3D7_1446900) was aligned to human autophagy-related protein 16-1 (ATG16L1, Q676U5) (alignment TM-score=0.47), a protein mapped to the GO term ‘positive regulation of autophagy’. The *P. falciparum* protein is possibly exposed on the surface of salivary gland sporozoites (Swearingen et al., 2016). ATG16L1 lacks a signal peptide and is mapped to the GO term ‘cytosol’ in Uniprot. From StringDB, we found that eight of 10 interacting proteins were associated with the Reactome Pathway ‘Macroautophagy’ (HSA-1632852) (**Figure S4**). Interestingly, while autophagy plays an important role in defence against intracellular parasites like *P. falciparum*, some parasites are known to manipulate this pathway for their own benefit; *P. berghei* growth is negatively affected when the human macroautophagy pathway is interrupted (Prado et al., 2015; Thieleke-Matos et al., 2016; Ghartey-Kwansah et al., 2020). Therefore, our results suggest that the *P. falciparum* protein mimics ATG16L1 to modulate the human macroautophagy pathway.

Additionally, four of these 145 P*. falciparum* proteins have been reported to be present in extracellular vesicles. Unlike the mimics identified in (Armijos-Jaramillo et al., 2021), our approach identified candidate mimics that can be identified using structure-similarity searches alone. These were the glideosome-associated connector (GAC, PF3D7_1361800), DNA/RNA-binding protein Alba 4 (ALBA4, PF3D7_1347500), high molecular weight rhoptry protein 3 (RhopH3, PF3D7_0905400), and pyridoxine biosynthesis protein PDX1 (PDX1, PF3D7_0621200). Interestingly, the parasite PDX1 protein was similar to the human copper homeostasis protein cutC homolog protein (CUTC, Q9NTM9) (alignment TM-score=0.69), which has copper binding activity (Li et al., 2010). While copper is an essential trace element for most organisms, excess copper is toxic for *Plasmodium* spp. (Rosenberg et al., 2006). This similarity to a copper binding protein suggests that PDX1 could function in copper sequestration in EVs.

## 4 Discussion

In this study, we present, to the best of our knowledge, the first genome-scale search of tertiary structural similarity between *P. falciparum* proteins and human proteins. We demonstrated the usefulness of our approach by showing that approximately 7% of the *P. falciparum* proteins had similarity to human proteins that could be detected only at the level of tertiary structure, and not primary sequence. We validated our approach on known mediators of host-parasite interactions and identified known similarities between CSP and thrombospondin-1 and AMA1 and the PAN protein domain in human plasminogen. Using available molecular and -omics datasets from PlasmoDB, we shortlisted a set of 44 instances of mimicry that are candidate mediators of host-parasite interactions, which improves our understanding of existing *P. falciparum*-human interactions.

We consider there to be six points to for the interpretation of the results of this analysis. First, the source of the structural model (PDB or AlphaFold) made a considerable difference (<50% agreement) for 10% of proteins. We emphasize that this is for AlphaFold structures that were filtered for high confidence; we anticipate that for lower confidence AlphaFold structures, an agreement with a PDB structure would be worse. Improvement in the AlphaFold software and its evolutionary models will lead to better structural models, but users must remain vigilant of accuracy metrics when using this promising resource. If a particular gene of interest has a protein structural model available in the PDB, we recommend incorporating it in the analysis. A recent notable study attempted to overcome this challenge by improving AlphaFold predictions for two parasites, *Trypanosoma cruzi* and *Leishmania infantum* (Wheeler, 2021). They attributed the poor accuracy of tertiary structure prediction for several parasite proteins to the low number of representative parasite sequences used to predict them and demonstrated that they could improve the AlphaFold predictions by increasing the number of parasite sequences used to model these structures.

Second, we noticed that the number of parasite proteins with structural similarity, but not sequence similarity, to human and negative control proteins was similar for most species studied. Some of these would be distant homologs which are not detected by sequence-based tools employed in this study. Indeed, the failure of homology detection has been shown to be one of the important reasons for the presence of lineage-specific genes (Weisman et al., 2020). In fact, multiple studies have demonstrated the effectiveness of using structure data to identify distant homologs that are missed by sequence-based approaches (Andorf et al., 2022; Monzon et al., 2022). Other proteins, however, would represent false positives of our approach based on our definition of ‘molecular mimics’, in which we define that a mimic confers benefit to the parasite. To this end, we used experimental data on *P. falciparum* from PDB to shortlist 44 proteins in *P. falciparum* that are structurally similar to human proteins and have a high probability of benefiting the parasite *via* its mimicry of a human protein. It is important to note that most parasites lack similar resources, making such an analysis challenging. This highlights the need to develop multi-omics resources, like PlasmoDB, for other parasites of global health concern.

Third, we discarded AlphaFold predictions with low prediction accuracy, so remove structures with a high level of intrinsic protein disorder. This is unfortunate, as protein-protein interactions are often mediated by disordered regions. For example, it has been proposed that viruses modulate host cellular processes by mimicking regions in disordered regions (Xue et al., 2014; Dolan et al., 2015; Garg et al., 2022). To potentially identify mimicry in disordered regions, it may be beneficial to consider a sequence *k*-mer based approach.

Fourth, the choice of structure aligner can impact the results. Foldseek, while being significantly faster than other available aligners, may trade off sensitivity (Holm, 2022; van Kempen et al., 2023). DALI, on the other hand, is arguably the most accurate but may not be suitable for large-scale structural similarity searches due to its longer run time.

Fifth, it is important to inspect the version of each protein sequence in the Uniprot database and its corresponding structure in the PDB and AlphaFold.The Uniprot database is updated routinely and identifiers assigned to a protein may change, the corresponding protein in the PDB or AlphaFold would probably not be updated as often as Uniprot.

And sixth, we acknowledge the need for validation. In this study, we identified *Plasmodium* proteins which structural resemble non-homologous human proteins. We use additional data to highlight those proteins which may be interacting with human proteins to the benefit of *Plasmodium*. The next step is validation, ideally through a biochemical assay, e.g., Yeast Two Hybrid or a mass spectrometry approach [reviewed in (Nicod et al., 2017)]. Computational approaches can be used to dock two proteins and predict their binding affinities. However, the accurate calculation of binding energies between two proteins is a laborious and error-prone process, lacking consistency between methods. For most of the predictions presented here, we have used AlphaFold models; a small change in side-chain orientation may be tolerated in structural alignments but can lead to a dramatic change in binding energies. We note that algorithms which refine structural models and use them for a range of purposes are being released at a frenetic rate. However, we remain cautious at introducing further technical uncertainty; a recent study showed that only 51% of PPIs could be corrected predicted from AlphaFold models (Bryant et al., 2022).

In conclusion, we present, to the best of our knowledge, the first genome-level search of tertiary structure similarity between *P. falciparum* and human proteins, leading to the identification of instances of molecular mimicry. Leveraging the extensive experimental data available for *P. falciparum* on PlasmoDB, we sought to provide biological relevance of the identified similarities. The catalogued list of 44 candidate mimics serves as candidates for experimental validation. It is worth noting that this list is a subset of the total identifiable mimics; containing those which could not be identified by commonly used sequence similarity approaches. Our findings contribute to a deeper understanding of molecular mechanisms underlying *Plasmodium*-human interactions and may have help guide vaccine and drug development against malaria.

## Data availability statement

The datasets presented in this study can be found in online repositories. The names of the repository/repositories and accession number(s) can be found in the article/**Supplementary material**.

## Author contributions

Conceived and designed the analyses: VM and JW. Performed the analyses: VM. Wrote the manuscript: VM and JW. All authors contributed to the article and approved the submitted version.
