# Supplementary material for: Proteome-wide comparison of tertiary protein structures reveals molecular mimicry in Plasmodium-human interactions
Source: Front Parasitol. 2023 Jun 15;2:1162697. doi: 10.3389/fpara.2023.1162697 (PMC11732093; doi:10.3389/fpara.2023.1162697)
Supplement: Supplementary file 1 [file DataSheet_1.pdf]

Supplementary Material

Proteome-wide comparison of tertiary protein structures reveals the role of molecular mimicry in *Plasmodium*-human interactions

Viraj Muthye[1,2], James D. Wasmuth,[1,2*]

[1]Wasmuth Laboratory, University of Calgary, Faculty of Veterinary Medicine, Calgary, Alberta, Canada

[2] Host-Parasite Interactions Research Training Network, University of Calgary, Calgary, Alberta, Canada

*** Correspondence:** Dr. James D. Wasmuth (jwasmuth@ucalgary.ca)

1 Supplementary Data

Supplementary Files S1-S6 and the Foldseek results for this study have been deposited in the Open Science Framework repository at Muthye, Viraj. 2023. 'MimicryMS.' OSF. February 6, 2023 https://osf.io/cusyg/ (DOI: 10.17605/OSF.IO/CUSYG)

2 Supplementary Figures and Tables

2.1 Supplementary Figures

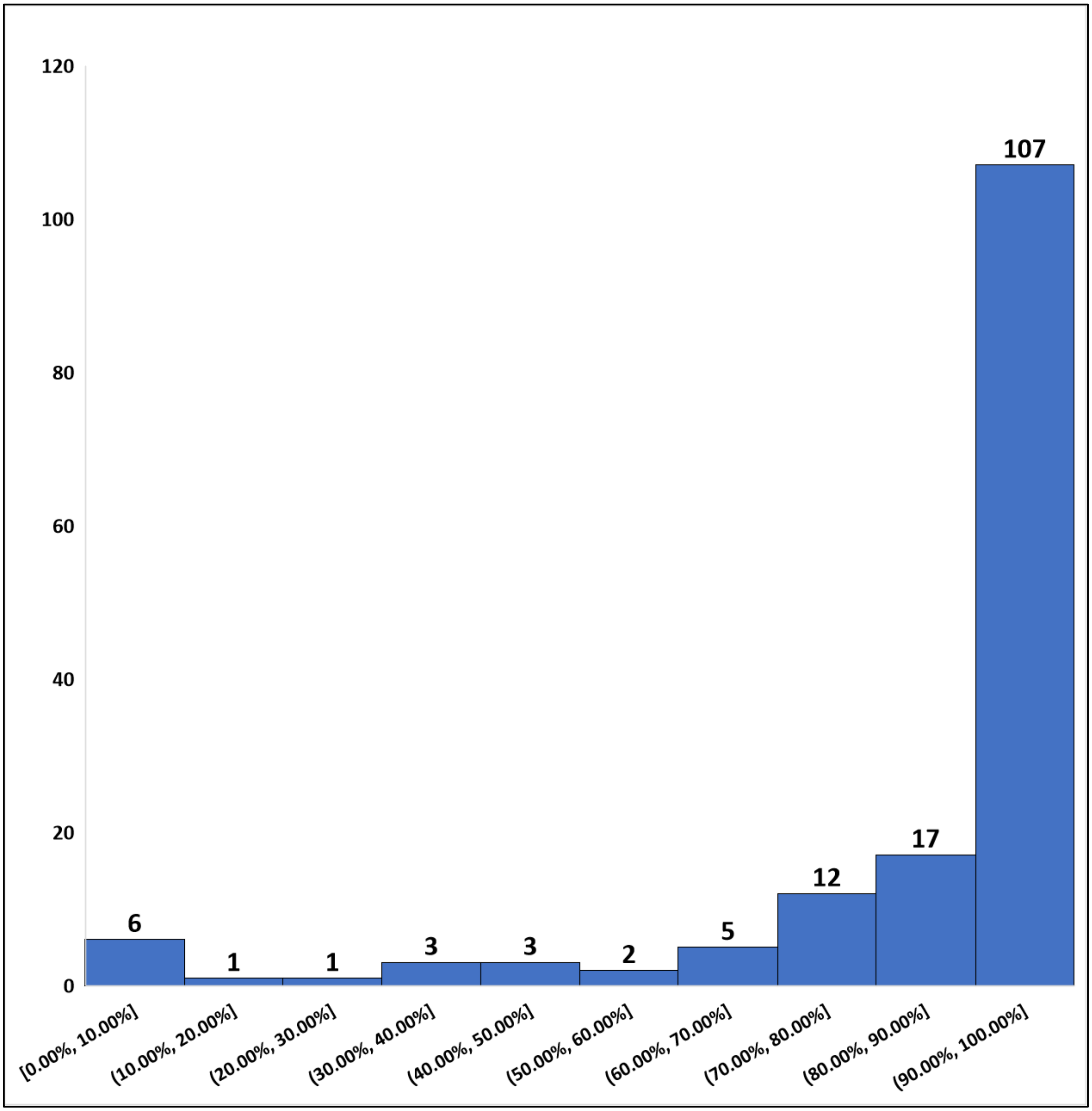

Figure S1 Overlap between the number of (host and control) structures aligned to the AlphaFold structures and the number of (host and control) structures aligned to both PDB and AlphaFold structures.

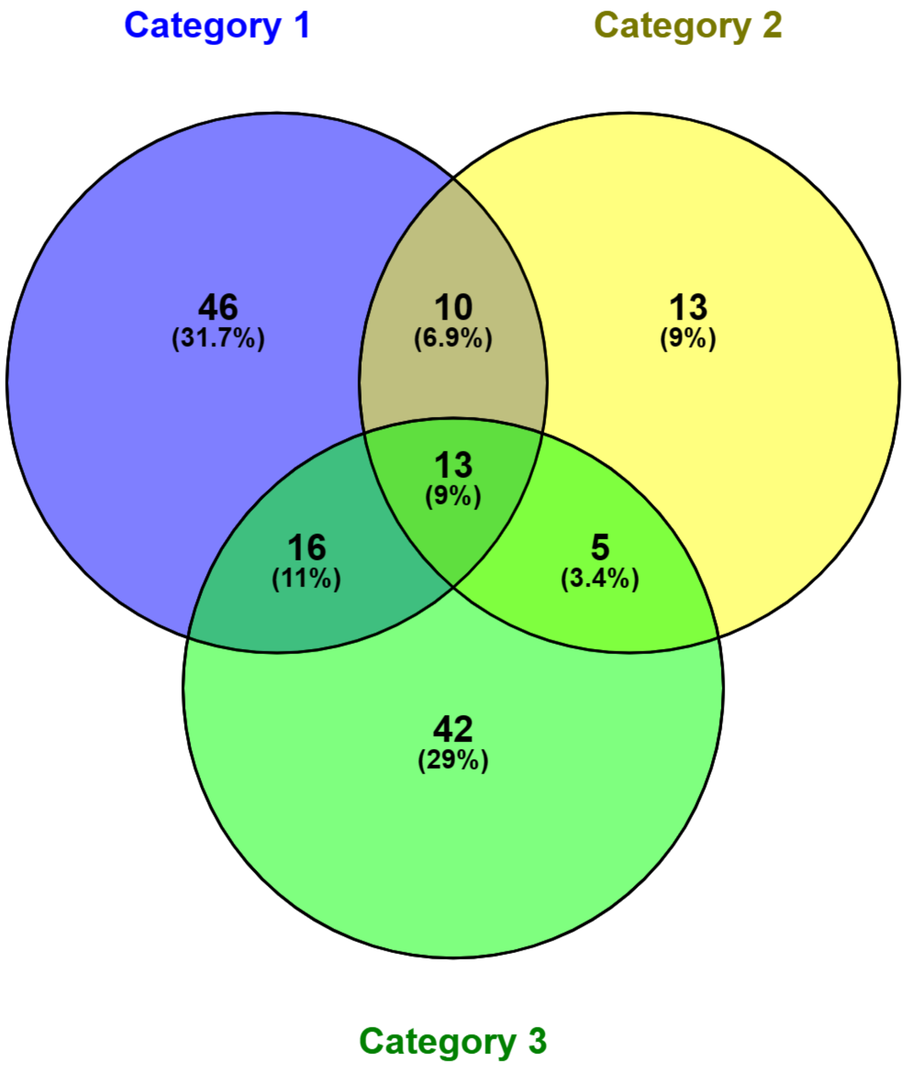

Figure S2 Distribution of the number of parasite proteins identified by three categories listed in Section 3.4.

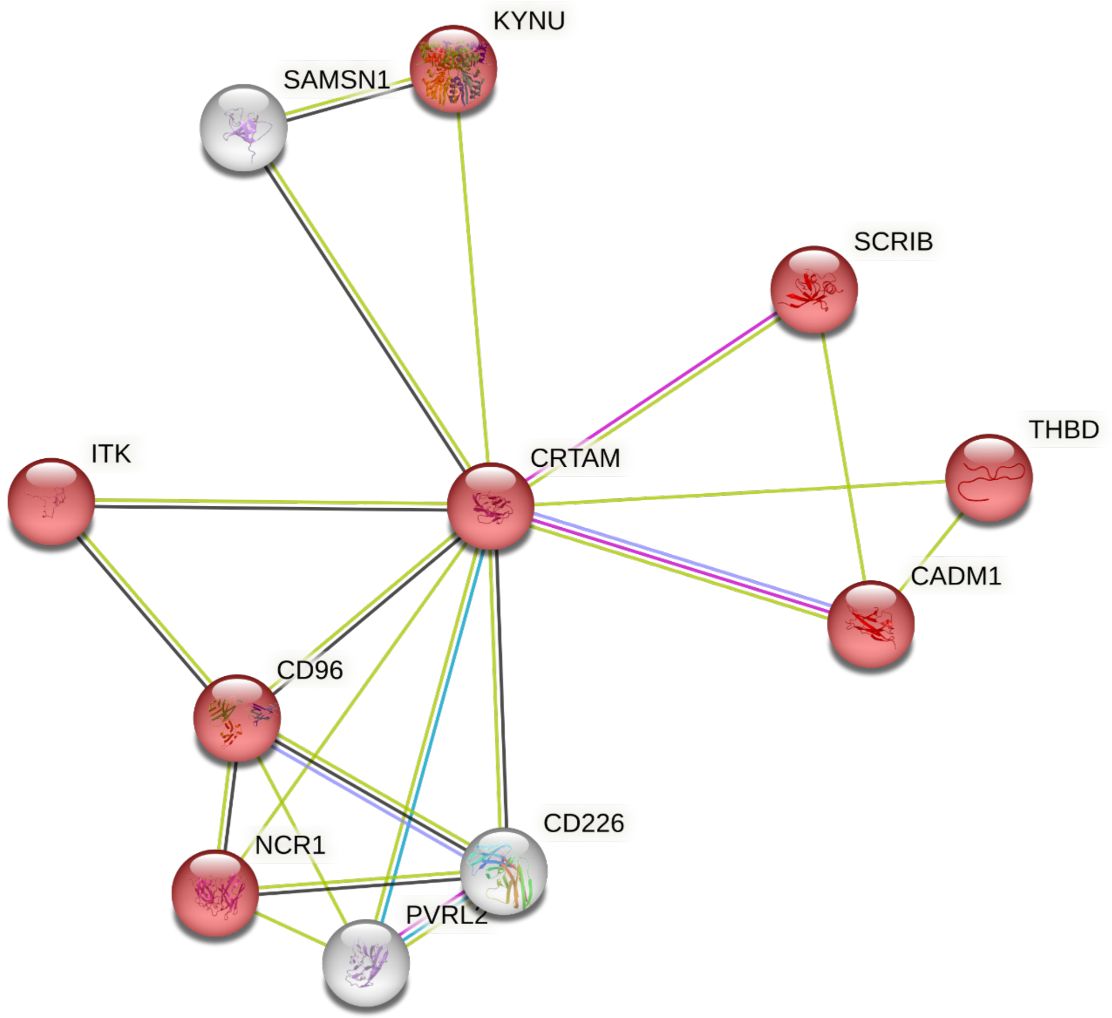

Figure S3 StringDB analysis of the human cytotoxic and regulatory T-cell molecule (CTRAM, O95727). Red proteins function in 'immune system process (GO:0002376)'.

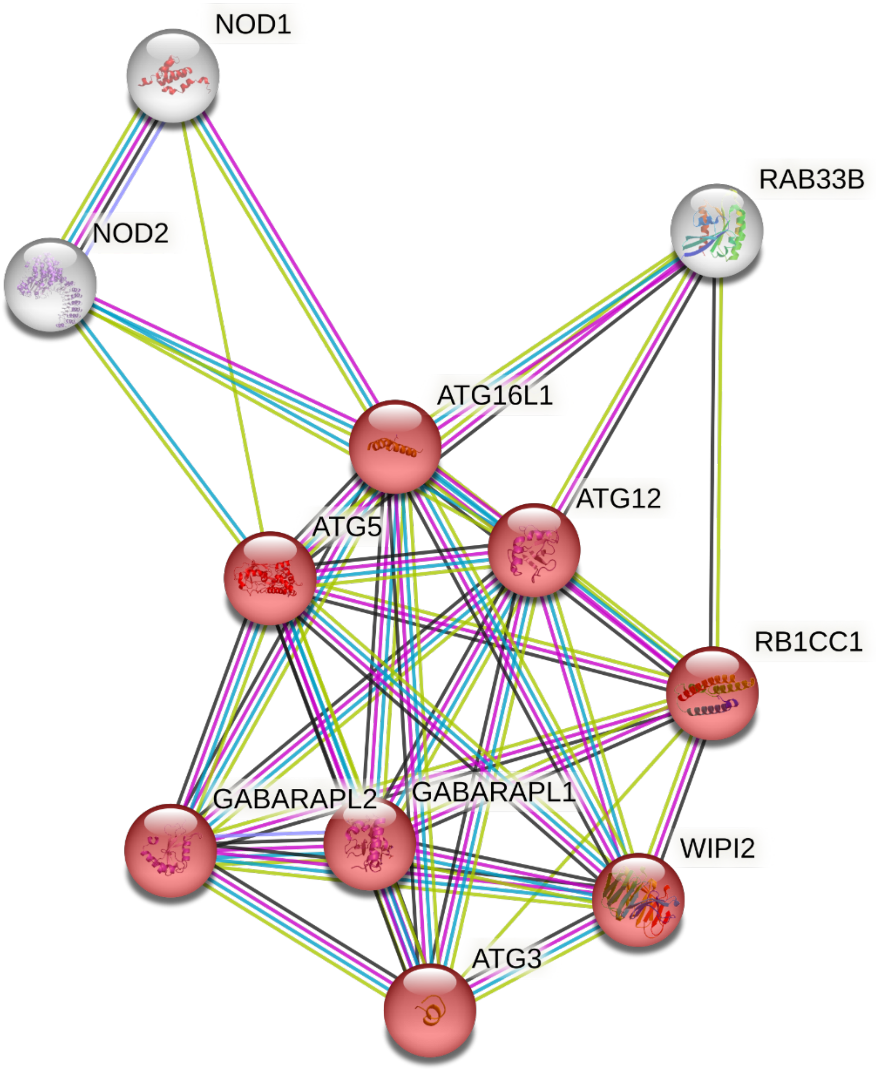

Figure S4 StringDB analysis of the human autophagy-related protein 16-1 (ATG16L1, Q676U5). Red proteins function in the Reactome Pathway 'Macroautophagy (HSA-1632852)'.
